# Attitudes toward ophthalmology as a prospective career among pre-clinical medical students in China

**DOI:** 10.1186/s12909-023-04518-4

**Published:** 2023-07-28

**Authors:** Yixin Yu, Yi Ding, Yannis M. Paulus, Haibo Jiang

**Affiliations:** 1 Eye Center of Xiangya Hospital, Central South University, Changsha, Hunan Province China; 2 Hunan Key Laboratory of Ophthalmology, Changsha, Hunan Province China; 3 Department of Ophthalmology and Visual Sciences, University of Michigan, Ann Arbor, MI USA; 4 Department of Biomedical Engineering, University of Michigan, Ann Arbor, MI USA

**Keywords:** Medical student, Medical specialty decision, Ophthalmology advising, Medical career advising, Survey of attitudes toward ophthalmology

## Abstract

**Background:**

A questionnaire was developed and administered to 450 medical students at the Xiangya Medical College, Central South University in Changsha, China to understand the attitudes among medical students in China toward different medical specialties and to find the factors that influenced their choice of career in ophthalmology.

**Participants:**

Fourth-year medical students in the five-year program and sixth-year medical students in the eight-year program.

**Methods:**

All the students were asked to rate the importance of nine possible factors in choosing a specialty as their vocation and their first ranked future specialty career choice.

**Results:**

When asked about the reasons for choosing to go to medical school, the top four reasons are the ability to help patients, interesting and challenging work, prestige, and job stability. When asked about the reasons for choosing a specialty, the top four reasons are the ability to find employment, financial reward, career upward mobility, and professional pressure. About the first career choice of the future specialty, for clinical medicine students, ophthalmology is the fifth ranked choice for clinical medicine students. 5.6% (five-year) and 3.4% (eight-year) of them choose ophthalmology as their top ranked specialty for their career. For anesthesia medicine and oral medicine students, most of them preferred to choose the same specialty as before. 1.5% (anesthesia) and 4.5% (oral) of them chose ophthalmology as their top ranked specialty.

**Conclusions:**

Medical students in China have numerous factors that motivate their choice in a specialty. Ophthalmology is the fifth ranked choice among clinical medicine students.

## Introduction

Vision impairment is a leading and largely preventable cause of disability worldwide. 32.4 million people were legally blind in 2010 globally; 17% of them lived in China [1]. With an increasing and aging population in China, the blind population may increase in the future. Cataract remains the leading cause of blindness among visually impaired people in the world, yet cataract blindness is reversible since surgical intervention can effectively restore visual impairment [2]. Cataract surgical rate (CSR) is defined as the number of cataract operations performed per million people in 1 year and is used as a proxy indicator of access to eye care services in a country [2]. In developed countries (such as the United States, Europe, Australia, and Japan) where avoidable causes of blindness are less common, the CSR ranges from 4,000 to 10,000. The CSR in China is significantly lower at 1,067. China’s cataract surgery rate is even lower than that of neighboring countries like India (5,600) and Vietnam (1,900) [2].

Several possible reasons exist for the large avoidable population with blindness in China. First, China has a large rural population with limited accessibility and affordability of eye care services. Almost 500 counties in China lack eye care services or an ophthalmologist [3]. Second, China has a rapidly aging population. Third, China has a shortage of skilled ophthalmologists. In 2012, the total number of ophthalmologists in China was 28,338 (20 ophthalmologists per 100 000 people) and only 36% of the ophthalmologists can perform surgery. In the United States, there are 59 ophthalmologists per 100,000 people, and 80% of them can perform surgery [4]. Even in some well-equipped hospitals, the utilization of the operation room for ophthalmology can be low because of the shortage of skilled ophthalmologists.

The training of skilled ophthalmic surgeons is an expensive and important element to reduce the prevalence of avoidable blindness and visual impairment in China [5]. To evaluate the number of ophthalmic surgeons, one needs to evaluate the pipeline for ophthalmic surgeons and how academic institutions determine the number and training of students each year. It is also critical to evaluate medical students’ attitudes towards ophthalmology and the factors that influence medical students’ future specialty choice.

In China, medical students enroll directly after high school rather than completing a 4-year college degree like in the United States. Thus, medical students in China choose their specialties at the completion of medical school at a relatively younger age. According to the American Association of Medical Colleges (AAMC), the mean age at matriculation of medical school in the United States was 22.6 years old in 2017 [6], whereas in China the mean age is 18.6 [7]. Medical students in China decide their specialty from career advisors, mentors, the environment of the school, their exposure to ophthalmology in their class, and their experience with ophthalmology instructors. Medical students in China can be divided into five-year degree programs and eight-year programs. Ophthalmology is taught during the fourth year (for five-year program) and the sixth year (for eight-year program) of medical school, which is the critical time for them to make career choices. There are five aspects according to previous research [8, 9] that influence their choice for their future career: ability to help others, interesting and challenging work, job stability, financial reward, and prestige. Currently many other aspects may also influence their specialty choice in China, including ability to find employment, good doctor-patient relationship, career upward mobility, and professional pressure. Understanding the attitude of medical school students is thus very important to recruit medical students to ophthalmology. We investigated nine attitudes toward different specialties among medical students in the medical school of Central South University, Changsha, China. We also investigate the experience of the students who selected to enter the field of ophthalmology. The goal of this study is to analyze the factors that influence the specialty choice by medical students in China.

## Materials and methods

### Study setting

In Central South University Xiangya School of Medicine, medical students are admitted directly from high school. The five-year program is divided into 2 years of basic science, 2 years of clinical medicine, and 1 year of internship. Five-year program students are divided into the departments of clinical medicine, anesthesiology, and oral medicine. The eight-year program is divided into 4 years of basic science, 2 years of clinical medicine, 1 year of internship, and 1 year of research. The inclusion criteria in our study were fourth-year five-year program students, including department of clinical medicine, anesthesiology, and oral medicine, and sixth-year eight-year program students, including eight-year program clinical medicine department. All of the students were enrolled after completing their rotation in ophthalmology. All the participants were informed the purpose of this study. Informed consent was signed and obtained from each individual. The questionnaire did not contain any identifying information about the individuals. Participation was totally voluntary. The exclusion criteria are the participants who declined to answer specific questions or left the questionnaire blank. The protocol was approved by the Central South University Institutional Review Board (IRB). All the participants were asked to complete a questionnaire involving their demographic characteristics, their attitudes towards different medical specialties, present life, future life plans, and attitudes towards ophthalmology and ophthalmologists. The survey took approximately 20 min to complete.

486 medical school students agreed to participate in the study. Some of the questionnaires were not completed, so the current study includes 450 students (92.6% of those who consented).

Questionnaire

The questionnaire consisted of six areas:

1. The social-demographic characteristics of the students, including age, gender, and the specialty they chose as their major.
2. The attitudes towards present life and the future career planning, which including 4 questions:
  1. Do you regret choosing medicine school? Yes/No.
  2. Are you satisfied with your present life? Yes/No.
  3. Does studying occupy most of your present life? Yes/No.
  4. What is your plan after graduation?
3. The intention to choose the various medical specialties (internal medicine, surgery, obstetrics & gynecology, pediatrics, infectious diseases, anesthesiology, dentistry, otolaryngology (ENT), dermatology, emergency medicine, psychiatry, and ophthalmology) after graduation. Each specialty was assessed by a scale with 1 (top choice), 2 (most likely), 3 (maybe) and 4 (unlikely).
4. The reasons for choosing to go to medical school and the reasons for choosing a future specialty. Students were free to choose the multiple reasons from nine possible factors we listed below that influencing the choice of a specialty as a vocation.
  - Being a doctor in this field leads to a stable life.
  - Being a doctor in this field can earn good money.
  - This is a job with prestige.
  - This is an interesting and challenging job.
  - Being a doctor in this field has enough ability to help patients.
  - Being a doctor in this field gets promotion very fast.
  - Professional pressure is relatively low in this field.
  - The doctor-patient relationship is good in this field.
  - Being a doctor in this field can find employment easier.
5. The attitudes towards different specialties (the same ones listed in item #3 above) in these nine factors that are listed above. Multiple-choice responses to each factor were defined as follows: 1 (strongly agree), 2 (moderately agree), 3 (moderately disagree), 4 (strongly disagree).
6. The impression for those entering the field of ophthalmology.
  What is the most important quality for an ophthalmologist?
  - Communication skills.
  - Surgical skills.
  - The ability to diagnose and treat diseases.
  - The ability to do the research.
  - The ability to do popular science.

## Results

### Demographic characteristics of medical students

The study (450 students) is composed of 46.7% male and 53.3% female students. The mean age of students was 21.8 years (standard deviation = 1.59, range 19–26). Among these 450 students in our sample, they are from four different specialties in their studies. The demographic characteristics of the subjects who completed the questionnaires are shown in Table 1.

**Table 1:** Demographic Characteristics of Medical Students (N = 450)

	Percentage (%)	Number
**Gender**		
Male	46.7%	210
Female	53.3%	240
**Age**		
19	0.2%	1
20–22	72.9%	328
23–26	26.9%	121
**Specialty**		
Clinical Medicine (five-year)	51.8%	233
Clinical Medicine (eight-year)	19.6%	88
Anesthesia Medicine	13.7%	62
Oral Medicine	14.9%	67

### Attitudes on the present life and the future career planning

When evaluating the attitudes on their present life (Table 2), 29.6% students regretted their decision to choose medical school, 32.7% of them were unsatisfied with their present life, and 89.8% reported their lives were occupied with studying. Among these students, eight-year program students tended to be more satisfied with their present life compared to others. When asked about their plan after graduation among eight-year program students, 96.6% of them had plans to be a doctor. However, our sample also included eight-year program students which would not have to get a master’s degree after their graduation. During the five-year program students (N = 362), 68.0% of them would like to further their studies after graduation instead of finding employment.

**Table 2:** Attitudes on the present life and the future career planning (N = 450)

	Percentage (%)	Number
Regret to choose medicine		
Clinical Medicine(8-year)	25.0%	22
Clinical Medicine(5-year)	27.0%	63
Anesthesia Medicine	41.9%	26
Oral Medicine	32.8%	22
Total	29.6%	133
Unsatisfied with present life		
Clinical Medicine(8-year)	26.1%	23
Clinical Medicine(5-year)	30.0%	70
Anesthesia Medicine	32.3%	20
Oral Medicine	50.7%	34
Total	32.6%	147
Studying occupies most of my life		
Clinical Medicine(8-year)	88.8%	79
Clinical Medicine(5-year)	88.4%	206
Anesthesia Medicine	93.5%	58
Oral Medicine	91.0%	61
Total	89.8%	404
Plan after graduation (N = 88, 8-year Clinical Medicine)		
Doctor	96.6%	85
Researcher	2.3%	2
Other	1.1%	1
Plan after graduation (N = 233, 5-year Clinical Medicine)		
Doctor	29.6%	69
Get master’s degree	69.1%	161
Researcher	0.4%	1
Other	0.9%	2
Plan after graduation (N = 62, Anesthesia Medicine)		
Doctor	27.4%	17
Get master’s degree	64.5%	40
Researcher	4.8%	3
Other	3.2%	2
Plan after graduation (N = 67, Oral Medicine)		
Doctor	29.8%	20
Get master’s degree	67.2%	45
Researcher	0%	0
Other	3.0%	2
Plan after graduation (N = 362, excluding eight-year program students)		
Doctor	29.3%	106
Get master’s degree	68.0%	246
Researcher	1.1%	4
Other	1.6%	6

### Top career specialty choice of medical students

**Fig. 1:**
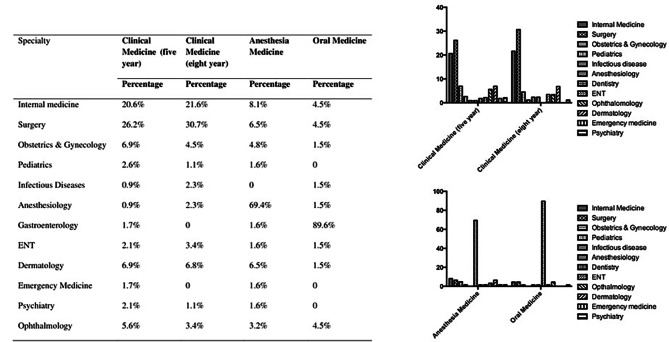
Top career specialty choice of medical students

Figure 1 demonstrates the top career specialty choice of medical students. For clinical medicine students, the most popular specialties are internal medicine and surgery, followed by obstetrics & gynecology and dermatology. Ophthalmology is the fifth ranked choice among clinical medicine students just below these four specialties. 5.6% (five-year) and 3.4% (eight-year) of them chose ophthalmology as their top career specialty choice.

For anesthesia medicine and oral medicine students, which have their specialty in the five-year program but can also choose a new specialty after graduation, most of them (69.4% anesthesia medicine students and 89.6% oral medicine students) preferred to choose the same specialty as before. For those who want to change their specialties, anesthesia medicine students chose internal medicine (8.1%) and surgery (6.5%), and oral medicine students chose ophthalmology (4.5%), internal medicine (4.5%), and surgery (4.5%).

### The reason for choosing to be a doctor, before and after four or six years of medical school

**Fig. 2:**
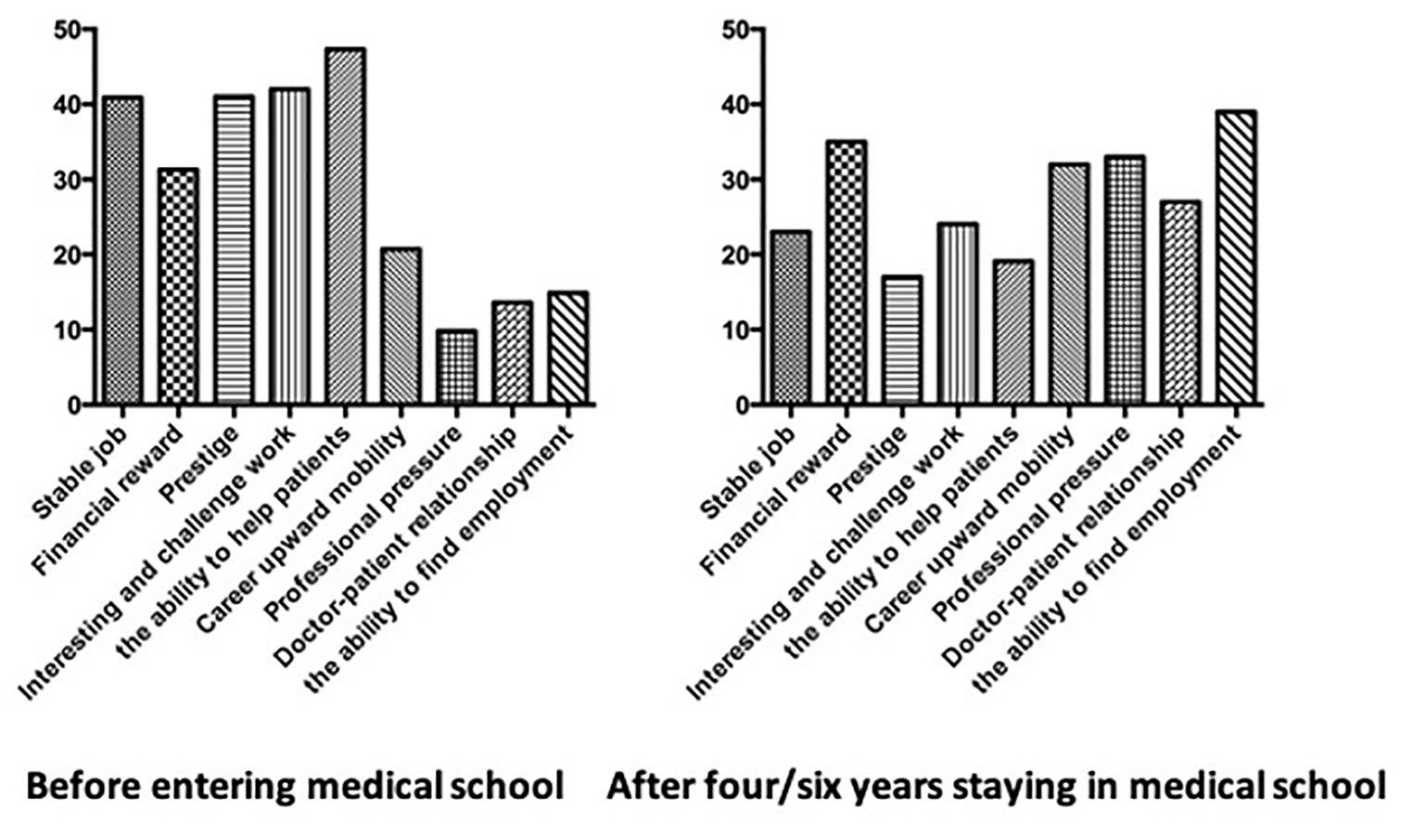
The reason for choosing to be a doctor, before and after four or six years of medical school

The choice to apply for medical school was made by the students immediately after graduation from high school. When asked about the reasons for choosing to go to medical school, the top four reasons are the ability to help patients, interesting and challenging work, prestige, and job stability. About the reasons for choosing a specialty, which the choice was made at the fifth (for five-year program students) or sixth (for eight-year program students) year of medical school, the top four reasons are the ability to find employment, financial reward, career upward mobility, and professional pressure (Fig. 2).

### The attitudes towards different specialties

**Fig. 3:**
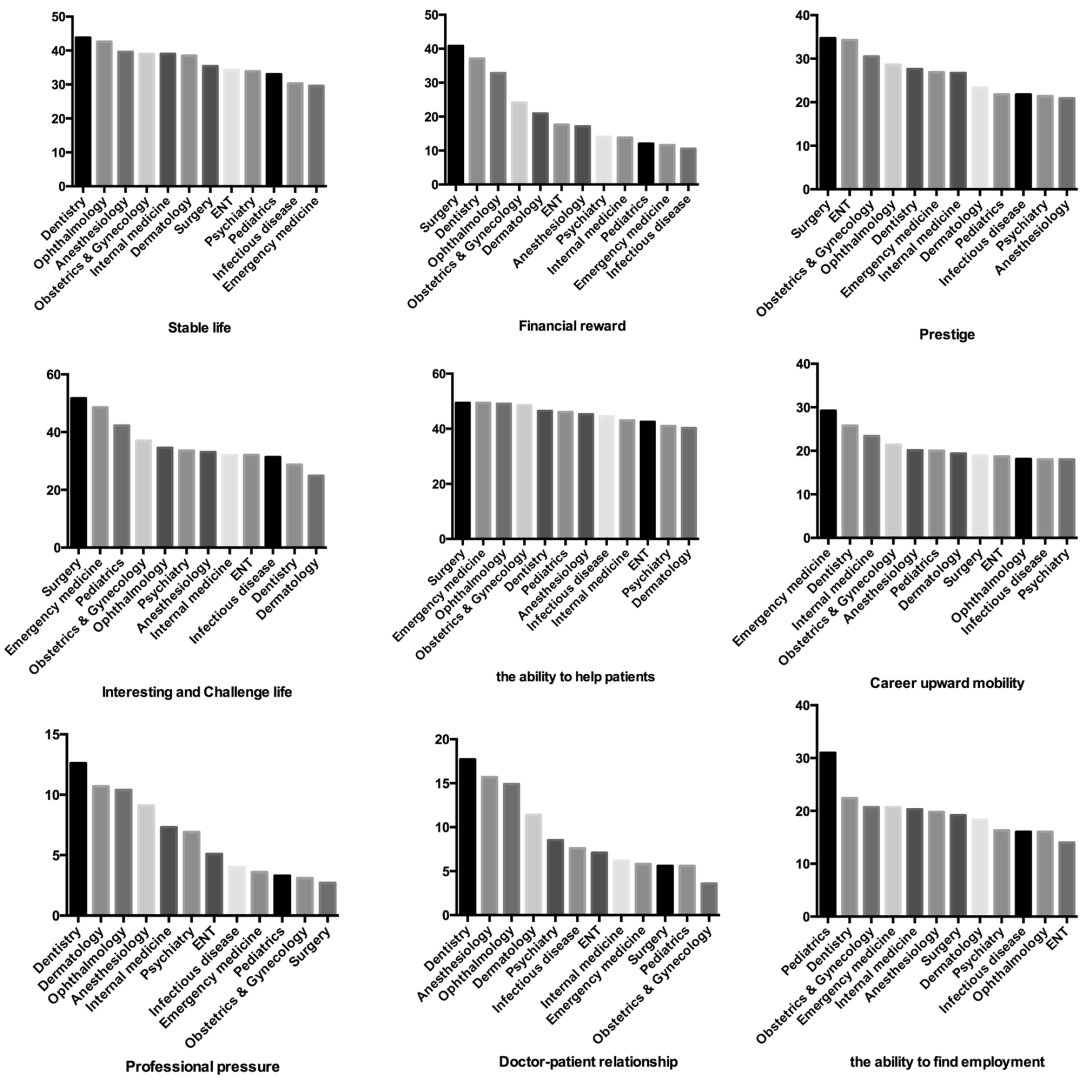
The attitudes towards different specialties

These results are rated by the percentage of students that totally agreed that a specific specialty offered a specific opportunity (Fig. 3). Students rated overall high scores in stable life, interesting and challenging work, and the ability to help patients. Students rated overall low scores in professional pressure and the doctor-patient relationship. These nine attitudes can be classified into subjective and objective attitudes. Stable life, prestige, interesting and challenging life, and the ability to help patients tend to more subjective. Ophthalmology receives a high-rating in these four subjective attitudes. In the objective attitudes, ophthalmology also received a high-rating on financial reward, professional pressure, and the doctor-patient relationship. But on the attitudes of the ability to find employment and career upward mobility, ophthalmology received very low-rating and is one of the lowest three specialties.

### Requirements for the ophthalmology class and ophthalmologic teachers

**Fig. 4:**
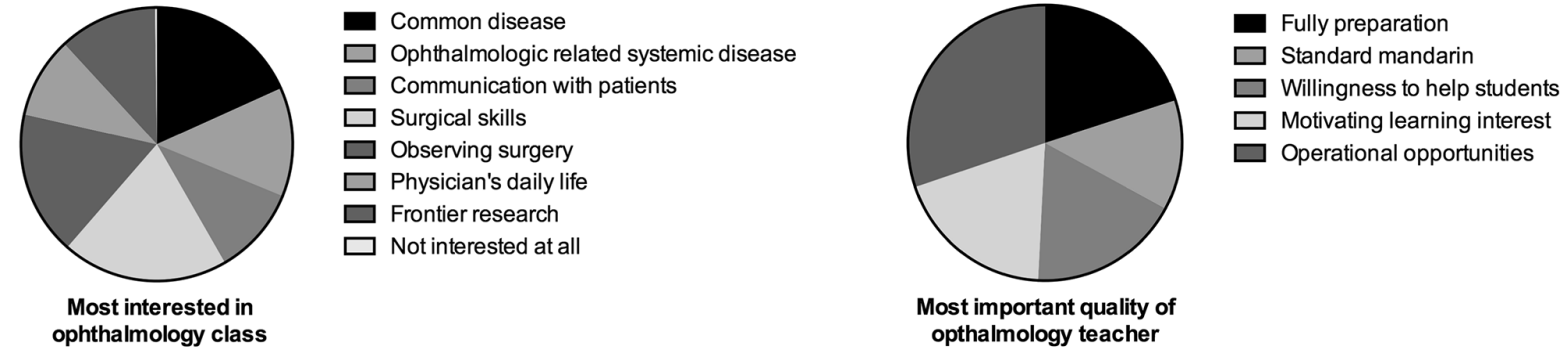
Requirements for the ophthalmology class and ophthalmologic teachers

For the ophthalmology class, students were more interested in operational skills (19.8%), common diseases (18.3%), and observing surgery (17.1%), followed by ophthalmologic related systemic disease (12.9%), cutting-edge research (11.7%), communication with patients (10.5%), and physicians’ daily life (9.6%). For the requirements for the ophthalmologic teachers, 30.2% of students preferred a teacher who was willing to give operational opportunities to the students, followed by being fully prepared for class (20.0%), motivating students’ learning interests (19.0%), willingness to help students (17.9%), and standard Mandarin communication (12.9%) (Fig. 4).

## Discussion

This study seeks to understand the attitudes and perceptions of medical school students in China to different specialties to determine the factors that lead students to select a particular specialty with a particular focus on the field of ophthalmology. According to the results (Table 2), 89.8% of students report spending most of their time studying with little time for rest. There is a saying that is very popular among Chinese medical students that, “Medical school is like repeating the life of your third-year high school for 5 times.” 29.6% of students regret their decision to enter medical school, and 32.7% of students are dissatisfied with their life recently. This negative attitude towards life likely reflects not only their current life in medical school but also the reports from the media regarding the real environment of being a doctor in China. The mean annual salary in 2015 of Chinese physicians, adjusted for purchasing power, was around U.S. $27,000, which is many times lower than the average physician salary in some developed countries [10]. Doctors in China also face considerable professional pressure. The demand for healthcare services in China is rising rapidly but the number of doctors has not increased accordingly [11]. Poor doctor-patient relationship is becoming a big problem. There were about 70,000 medical conflicts and more than 10,000 violent attacks on healthcare workers in China in 2013 [12]. The incidence of anxiety and depressive symptoms among physicians in China was higher than that in other countries [13].

In this study, we investigated 362 students in the 5-year program. We found that most (68.0%) of them will choose to further their study in the future (Table 2). Most provinces in China recently required every physician to pass the Standardized Resident Training (SRT) program before finding employment. This takes 3 years, which is the same amount of time as getting a master’s degree. Most students will choose to pursue further training in medical research in this study for a greater chance of publishing and easier access to a doctorate degree, which can lead to a more promising future. The students can also go through the first-level of SRT during these 3 years. After getting the master’s degree or the doctorate degree, most of the students will choose to work at the top ranking hospitals in cities rather than the rural hospitals, which will perpetuate the situation of the dearth of ophthalmologists in rural and remote places.

The reasons students choose to be a doctor is demonstrated in Fig. 1. Most students chose to enter medical school for idealistic reasons like the ability to help patients, interesting and challenging work, and prestige. But after four/six years of medical school, the reasons for choosing a specialty become more realistic, including the students considering more career upward mobility, the ability to find employment, and financial reward. All these results demonstrate that the medical school students have changed significantly in their mindset during these years. After four years in medical school, students consider more about their future life. This change is inseparable from the burden of studying in school, and media reports about the working environment of doctors in China.

Few studies [8, 14, 15] have focused on these nine attitudes towards ophthalmology as students decide their future career, especially the attitudes on the ability to find employment, career upward mobility, professional pressure, and doctor-patient relationship. Our finding (Table 1) indicated that for clinical medicine students, 5.6% (five-year) and 3.4% (eight-year) of them ranked ophthalmology as their first choice career. For the anesthesia and oral medicine students, 69.4% anesthesia medicine students and 89.6% oral medicine students preferred to choose the same specialty as before. Most students who have a specialty (e.g., anesthesia medicine and oral medicine) will choose to continue their specialties after graduation. This suggests that encouraging medical schools to launch ophthalmology as a specialty could recruit more future ophthalmologists.

Towards these nine attitudes (Fig. 3), ophthalmology seems to receive a high rating on the ability to help others, interesting and challenging work, stable job, financial reward, prestige, good doctor-patient relationship, and professional pressure. But for the ability to find employment and career upward mobility, students expressed a negative assessment about ophthalmology. These were very important for the students in making the decision for their future specialty. Even though their assessment on the other factors was positive, the students appeared to face realistic problems which reduced their interest in entering the field of ophthalmology.

This survey reflects the students’ perception of reality. Students likely have misunderstandings on each specialty. Students perceived that career upward mobility is comparably slow for ophthalmologists or possibly for all the surgeons in China. In this study, surgical opportunities and observing surgery are especially important factors that students took into consideration when choosing to be ophthalmologists (Fig. 3). It is reported [16] that most surgeries in large Chinese hospitals are done by a small group of senior doctors. Less than 5% of surgeries were performed by doctors under the age of 43. A study showed that mainland Chinese ophthalmology residents had undertaken a median of 0 independent cataract operations during their training, compared with 100 cases for trainees in the Hong Kong Special Administrative Region over a similar period [17]. In the United States, ophthalmology residents are required to complete a minimum of 86 cataract surgeries as primary surgeon to complete their residency [18].

China is characterized by extreme imbalance and differences in regional development. The geographic and demographic distribution of doctors in China is unequal. One study [19] showed that the prior medical system reforms with price regulation in China drove medical staff and patients to larger hospitals. This results in congestion in larger hospitals and idle resources in smaller hospitals. In this study, most (70%) of students will choose to further their study to get a master’s degree or even a doctorate degree. Most of them probably won’t choose to go to the smaller hospitals to work. Since 60% of the Chinese population lives in rural areas [20], the prevalence of visual impairment and blindness is high in rural populations [11]. But the hospitals in rural locations often cannot attract ophthalmologists to work there, and there is less need for ophthalmologists in hospitals in urban places.

About the financial reward for Chinese doctors, this aspect is always perceived as a key factor affecting job satisfaction. The salary of Chinese physicians is comparably lower than physicians in America, Canada, and some European countries. A survey [21] in China showed that 78% of physicians who wouldn’t want their children to be physicians ranked low salary and long work hours as the top 2 reasons. Also, in our study, financial reward is a priority factor that influences the students’ choice for a future specialty. A report [10] of the salary of physicians in different specialties in China revealed that the three highest income specialties are ophthalmology, otolaryngology, and dentistry. In our study, students perceive them to be surgery, dentistry, and ophthalmology. The three lowest income specialties in China are pediatrics, internal medicine, and emergency medicine. In our study, students perceive them to be infectious diseases, emergency medicine, and pediatrics. From this comparison, students’ attitudes closely mirror reality. Ophthalmology has a great advantage on this aspect. However there is significant regional and district differences in salary, and the mean salary of Chinese physicians in the district in which this study was conducted is lower than other districts [10].

Recently, numerous reports and debates around violence in Chinese healthcare settings are taking place [21, 22]. According to a survey [23] by the Chinese Hospital Association between December 2012 and July 2013, hospital violence has increased during the past 5 years. 96% of healthcare workers have been abused or injured in 2012, and 39.8% of them planned to give up the medical profession or switch to another profession.

Regarding perceptions of stable life, prestige, interesting and challenging life, and the ability to help patients, ophthalmology receives a high-rating on these four attitudes. In a study [24] in America, the top three annual specialties for work hours are surgery, obstetrics & gynecology, and internal medicine; the bottom three are dermatology, emergency medicine, and psychiatry. Medical practices are different in China so this likely does not represent the situation in China. Teachers can make their class more attractive to students by increasing opportunities to enter the operation room and observing surgery and operating under supervision (Fig. 4). This could recruit more promising students to select the field and cultivate more future ophthalmologists and physicians to treat ocular diseases.

## Conclusion

This study evaluates the attitudes among medical students in China toward different medical specialties to find the factors that influenced their choice of career in ophthalmology. Based on these findings, to attract more potential ophthalmologists, medical schools could launch ophthalmology as a specialty, teachers could make their class more attractive and make some changes according to the requests from students like increasing the opportunities to enter the operating room, observe surgery, and operate under supervision. Other areas that could be addressed to improve interest in ophthalmology include low salary, limited employment opportunities for ophthalmologists, and the shortage of the eye care centers in rural places in China.

## Data Availability

All data generated or analyzed during this study are included in this published article.
